# Intelligence

**Published:** 1830-09

**Authors:** 


### INTELLIGENCE.

### MEDICAL SCHOOL, KING'S COLLEGE, LONDON.

The following appointments have been made: Mr. Mayo, professor of Anatomy and Physiology; Mr. Green, of Surgery; Dr. Hawkins, of Medicine; Dr. Bissett Hawkins, of Materia Medica. The chairs of Chemistry and Midwifery are not yet filled. The Demonstrator, we understand, will be appointed at the recommendation of the Professor of Anatomy.

King's College will open in October 1831: In the interval, Mr. Mayo will continue to deliver lectures at Great Windmill street. The noble museum room in Great Windmill street, which has contained in succession the collections of Dr. Hunter, Dr. Baillie, Mr. Wilson, and Mr. Bell, is likely to be occupied, during the ensuing winter, by the museum of King's College. The latter, it is said, will be commenced by the purchase either of Soemmerring's collection or of Mr. Mayo's, which, though small, contains several of the rarest and most beautiful specimens in London.

The school of Windmill street will probably not be closed, when Mr. Mayo leaves it; but is likely to fall into the hands of a zealous and talented pupil of Mr. Mayo's and Mr. Brodie's. We conclude, therefore, that Mr. Mayo's pupils will have the option, when King's College opens, either of following him thither, or of remaining to pursue their studies under his successor in Windmill street.

---

Dr. James Johnson has been appointed physician extraordinary to the King.

Mr. Arnott has been appointed surgeon extraordinary to her Majesty. We stated by mistake in our last Number that Mr. A. was attached to the medical establishment of the King.

Mr. Craddock is appointed apothecary to his Majesty's household.

The appointment of Dr. Warren as physician extraordinary to the King, has been cancelled at his own request.

His Majesty has been graciously pleased to bestow on Sir HENRY HALFORD the Grand Cross of the Guelph.

Sir GILBERT BLANE has resigned his appointment as physician to his Majesty, in consequence, it is said, of the place in which his name appears in the list published in the official Gazette.

---

HENRY EARLE, Esq. surgeon to St. Bartholomew's Hospital, has been elected one of the Council of the Royal College of Surgeons.

---

Mr. HOWSHIP will commence his next course of Surgical Lectures at his house, on the first Monday in October, at eight in the evening. The weekly examination of the students will be held every Saturday evening, from nine to ten o'clock.

---

Mr. W. B. LYNN has addressed a letter to the Royal College of Surgeons, stating that he "renounces all intention and desire" to become one of their body, in consequence of his friend, Mr. J. R. ELMORE, having been passed over at the recent election of a member. That Mr. Lynn had the right to remonstrate with the College for the act of injustice which he assumes them to have committed, we do not deny; but we regret, for his own sake, that his letter was not written with greater respect and temperance. The step which Mr. L. has taken appears to us altogether premature and injudicious. Had he waited till he himself had been elected one of the Council, he would have escaped all suspicion of declining an honour from the fear that he might not have the opportunity of accepting it.

---

### BIBLIOGRAPHICAL NOTICES.

### LIFE OF DR. FOTHERGILL.

The father of John Fothergill was a member of the estimable society of Quakers, and resided at Carr-End, in Yorkshire, the family estate of a preceding generation; where this excellent man was born in the year 1712. He was one of many children, a circumstance which is generally, and with justice, considered favorable to the developement of talents. His chief literary education was imbibed in the school of Jedburgh, in Yorkshire, a seminary which has acquired both classical and mathematical reputation. About the age of sixteen, he was apprenticed to Mr. Bartlett, an eminent apothecary at Bradford, who had previously been the master of Dr. Hilary. His sagacity and assiduity soon induced his intelligent preceptor to permit him to visit and prescribe for his patients. On the expiration of his term, he repaired to Edinburgh, at a period when the professorial chairs were occupied by Monro, Alston, Rutherford, Sinclair, and Plummer, all students of the Boerhaavian school, and whose merits have been recorded, by Fothergill himself, in an account which he published, in after-life, of Dr. Russell, his contemporary and associate. The eminent Monro discovered the powers of his pupil, and urged him to reside sufficiently long to obtain the doctorate—for till then he had only intended to qualify himself for the profession of an apothecary. He used to take notes of the heads of each lecture, and on returning to his lodging, translated into Latin those which had been given in English; he then carefully consulted and compared the opinions, both of the ancients and the moderns, on the subject of the lecture, to which he added such remarks upon each as his reading and reflection suggested. In his clinical studies he followed a similar plan; when any case occurred to fix his attention, he examined the various authorities which bore upon the point and formed a comparative result from their evidence and opinions. Many years afterwards he recommended this method to Dr. Lettsom, in a letter which concludes by enforcing "the careful perusal of Hippocrates, and also of Aretæus and Celsus; one can never be too well acquainted with the knowledge contained in the first, nor with the elegant expressions of the last." These modes of study are not peculiar to

Fothergill, but they are important to remark in tracing the steps by which an obscure man attained independence and distinction, and strengthen the evidence of the efficacy of earnest reading in a profession which is by many supposed to depend for success on natural abilities, or worldly industry, or mere personal observation; whereas, none more requires all the assistance that can be derived from the experience of others. Monro, in the fourth edition of his Osteology, which appeared in 1746, acknowledges the aid which he had obtained from his young pupil. In addition to his other occupations, Fothergill preserved a diary of his actions and occurrences, in Latin. He graduated in 1736, and chose *the use of Emetics* for the subject of his thesis. He now came to London, the scene of all his subsequent eminence, and frequented the wards of St. Thomas's Hospital, where his application was equally unremitting. In 1740 he accompanied some friends on a short excursion to the continent, of which a brief sketch remains in a Latin letter addressed to his friend Dr. Cuming, of Dorchester. On his return he settled in Gracechurch street, and in 1746 became a licentiate of the College of Physicians.

The fertility of his mind now began to evince itself in some detached essays, among which we remark one "On the Origin of Amber," his observations on the Manna Persicum, and more particularly those "On a case of recovering a Man dead in appearance." It is possible that these last may have had some share in contributing to the establishment and regulations of the Humane Society, which was several years after founded by the exertions of Dr. Hawes and others.

A large practice rapidly rewarded the pains which he had bestowed on his education: as to his pecuniary means or early patrons little information remains, but it is certain that he acquired employment more early than the generality of his brethren: it is probable that the religious community of which he was a worthy member contributed something to the rapidity of his advancement. Nothing (says Dr. Lettsom) hurt his feelings more, than an estimate of the medical profession, formed upon lucrative advantages; he was ever averse to speak of his pecuniary emoluments: "My only wish," he declared, "was to do what little business might fall to my share as well as possible; and to banish all thoughts of practising physic as a money-getting trade, with the same solicitude as I would the suggestions of vice or intemperance." These were not mere words of parade: when the success of his practice had elevated him to the summit of his career, he still professed, and acted upon, the same generous reasoning. "I endeavour," says he, in a letter written several years afterwards, "to follow my business, because it is my duty, rather than my interest; *the last is inseparable from a just discharge of duty*; but I have ever wished to look at the profits in the last place, and this wish has attended me ever since my beginning." In another place he remarks, "I wished most fervently, and I endeavour after it still, to do the business that occurred, with all the diligence I could, as *a present duty,* and endeavoured to repress every rising idea of its *consequences* ........ such a circumscribed unaspiring temper of mind, doing every thing with diligence, humility, and as in the sight of the God of healing, frees the mind from much unavailing distress, and consequential disappointment." These familiar effusions of a spirit which cannot be accused of imbecility or apathy, are the more interesting, because few of the distinguished members of the medical profession have bequeathed to us so explicit an avowal of the motives which animated or sustained them in one of the most rugged roads of human travel.

In 1748, Fothergill raised his reputation to a great height, by his "Account of the Sore Throat attended with Ulcers; a disease which hath of late years appeared in this city, and in several parts of the nation." A disorder of this kind had lately caused much havoc, and not least among the higher orders of society. Two nephews of the Duke of Newcastle had fallen victims to it, and its progress excited great alarm. Here Fothergill found an opportunity of bringing his penetration to bear on a topic of new and immediate interest, and he availed himself to the utmost of the favorable moment. He had observed that the methods of cure usually resorted to, such as bleeding, purging, and the medicines daily employed to remove inflammations, in general produced an injurious effect in this epidemic. It was confounded in ordinary practice with the common sore throat, or inflammation of the tonsils. Our author carefully distinguished the variety in the nature of the complaint, and in the progress of the symptoms, which were here usually of the typhoid kind, and presented a disposition to gangrene in the parts affected. His practice was temperate, yet not guided by any exclusive views; even gentle purgatives he found injurious, but clysters might be given in case of constipation. A gentle emetic was often prescribed; gargles, bark combined with the mineral acids, and various stimulants, formed the basis of the treatment. The Spanish physicians had pursued a somewhat similar plan in the visitations which they had experienced of this complaint. Fothergill uses the following expressions in summing up this subject: they will afford a specimen of his pathology. "The cause of this tendency (to putrefaction) is a putrid virus, or miasma sui generis, introduced into the habit by contagion; principally by means of the breath of the person affected. This virus, or contagious matter, produces effects more or less pernicious, according to the quantity and nature of the infection, and as the subject is disposed to receive or suffer by it. Putrefactive and malignant diseases, in common, admit of the most sensible and secure relief from discharges of the peccant matter, either upon the skin in general, or on particular parts of the body. The redness, and cutaneous efflorescence, in the present case, may be considered as an eruption of the like nature, and therefore to be promoted by such methods as have proved successful in similar diseases. A cordial, alexipharmic, warm regimen has been found by experience to be of the most use in such cases." Called in on this occasion by many of the first families in the metropolis, he improved the opportunity so well, that he soon acquired a large income. The generality of medical men usually entertain a particular affection for some one or more branches of their professional studies, and the public has gained considerably by this occasional preference, which, while it does not necessarily diminish the attention of the individual to the ultimate object of his science, is sometimes the source of discoveries or institutions important to the community at large. Chemistry and botany were the favorite objects of his hours of relaxation or retirement. At Upton, near Stratford in Essex, he purchased an extensive estate, and furnished a noble garden, whose walls enclosed five acres, with a profusion of exotics, which he spared no pains in collecting. "At an expense seldom undertaken by an individual, and with an ardour that was visible in the whole of his conduct, he procured from all parts of the world a great number of the rarest plants, and protected them in the amplest buildings which this or any other country has seen. He liberally proposed rewards to those whose circumstances and situations in life gave them opportunities of bringing hither plants which might be ornamental, and probably useful to this country, or her colonies; and as liberally paid these rewards to all that served him. If the troubles of war had permitted, we should have had the Cortex Winteranus, &c. &c., introduced by his means into this country; and also the Bread-Fruit, Mangasteen, &c., into the West Indies. For each of these, and many others, he had fixed a proper premium. In conjunction with the Earl of

Tankerville, Dr. Pitcairn, and myself, he sent over a person to Africa, who is still employed upon the coast of that country, for the purpose of collecting plants and specimens. Those whose gratitude for restored health prompted them to do what was acceptable to their benefactor, were always informed by him, that presents of rare plants chiefly attracted his attention, and would be more acceptable to him than the most generous fees. How many unhappy men, enervated by the effects of hot climates, where their connexions had placed them, found health on their return home at that cheap purchase! What an infinite number of plants he obtained by these means, the large collection of drawings he left behind will amply testify; and that they were equalled by nothing but royal munificence, at this time largely bestowed upon the Botanic gardens at Kew. In my opinion, no other garden in Europe, royal or of a subject, had nearly so many scarce and valuable plants. That science might not suffer a loss, when a plant he had cultivated should die, he liberally paid the best artist the country afforded, to draw the new ones as they came to perfection; and so numerous were they at last, that he found it necessary to employ more artists than one, in order to keep pace with their increase. His garden was known all over Europe, and foreigners of all ranks asked, when they came hither, permission to see it; of which, Dr. Solander and myself are sufficient witnesses, from the many applications that have been made through us for that permission."

The account which we have here given is the more honourable to Fothergill, because it comes from the pen of a distinguished individual, who was not merely an excellent judge of horticultural pursuits, but the patron of all that was useful to his country and his age,—the late Sir Joseph Banks. A winding canal, in the figure of a crescent, formed this garden into two divisions, and occasionally opened on the sight, through branches of rare shrubs, which lined the walks on its sides. In the midst of winter, when the earth was covered with snow, evergreens were here clothed in full verdure. Without exposure to the open air, a glass door, from the mansion-house, gave entrance into a suite of hot and green house apartments, nearly 260 feet in extent, and containing above 3400 distinct species of exotics, whose foliage formed a beautiful contrast to the shrivelled natives of colder climes. In the open ground, about 3000 distinct species of plants and shrubs bloomed on the return of summer.

Such displays were not in him the effect of ostentation; he had always in view the enlargement and elevation of his own mind, and the prospect of multiplying the enjoyments of his fellow-beings. He formed an useful exchange of the productions of the earth. From America he received various species of catalpas, kalmias, magnolias, firs, oaks, maples, and other valuable productions, which became inhabitants of his own soil, and some of which were capable of being applied to the most useful purposes of timber; and he transported, in return, the green and bohea teas from his garden at Upton, to the southern part of that continent. He endeavoured to improve the growth and quality of coffee in the West Indian islands; and procured from China the bamboo-cane, with a view of naturalizing it in our intertropical possessions. He made many efforts to introduce the cinnamon-tree into the West Indian colonies. He attempted to procure the tree which affords the Peruvian bark, and is said to have, at length, so far succeeded, as to have had one plant in his garden. To two captains of ships he offered each a reward of one hundred pounds for a plant in vegetation of the true Winter's bark (*Winterana aromatica*). But his attention was not confined to the vegetable kingdom. Da Costa is indebted to him for many valuable remarks inserted in his History of Shells, of which Fothergill possessed the best cabinet in England, next to that of the Duchess of Portland. His collection of minerals was more rare than extensive; and the gratitude of his numerous friends had supplied him with many curious specimens of the animal world. He afforded employment to many artists, in directing the delineation of various productions of Nature, which were too bulky to transport, or too perishable to preserve: twelve hundred of such drawings are said to have been in his possession, and were purchased, at his death, for the Empress of Russia, at the price of 2300l. His collection of natural history was purchased, on his decease, by the eminent Dr. William Hunter, and is, probably, at this moment, to be found, in part, in the museum which that distinguished physician bequeathed to the University of Glasgow, after having vainly solicited the ministers of the time to enable him to establish one in London.

He maintained a frequent correspondence with North America, where his father and brother had spent many years in the service of religion. Several families are said to have even crossed the Atlantic, with a view of placing themselves under his care. He earnestly laboured to promote the improvement of rural economy and of commerce in that part of the world. In conjunction with his friend, Peter Collinson (a name upon which we should be glad to dwell more at length), he encouraged the cultivation of the vine there; and still more usefully strove, with others, to abolish the *slave-trade* among his own brethren—an object which they at length had the happiness to accomplish.

Charity was the predominant feature in Fothergill's character; that beautiful quality which many find so difficult to imitate, and which, in most minds, is a flower the slowest to blossom, and the earliest to decay. Few names on the record of biography will bear comparison with him in this respect. We do not know whether this noble characteristic was in him the result more of an original tenderness of disposition, or of self-discipline and principle; it seems probable that the study of our Divine Revelation had opened this plenteous fountain of beneficence in a mind not naturally of an enthusiastic temperament. When, during the summer, he retired to Lea-Hall, in Cheshire, he devoted one day in every week to attendance at Middlewich, the nearest market-town, and gave his gratuitous advice to the poor. He assisted the clergy, not merely with his advice, but on numerous occasions with his purse: on one occasion he was reproved by a friend for his refusal of a fee from a person who had attained a high rank in the church: "I had rather," replied the doctor, "return the fee of a gentleman with whose rank I am not perfectly acquainted, than run the risk of taking it from a man who ought, perhaps, to be the object of my bounty." When he paid his last visit to patients in decayed circumstances, it was not unusual with him, under the appearance of feeling the pulse, to slip into their hand a sum of money, or a banknote; in one instance, this mode of donation is said to have conveyed one hundred and fifty pounds. To the modest or proud poverty which shuns the light of observation, he was the delicate and zealous visitor; in order to preclude the necessity of acknowledgement, which is often painful in such minds, he would endeavour to invent some motive for his bounty, and hence afford to the receiver the pretensions of a claim, while the liberal donor appeared to be only discharging a debt. Rarely was any subscription commenced on whose list the name of Fothergill did not stand foremost. When the success of our arms had filled our prisons with foreign captives, he was appointed member of a committee which superintended the sums raised for their relief; and it should be stated, to the honour of his community, that above one fourth of the whole subscription was contributed by the Quakers, who then scarcely formed the two hundredth part of the nation. To Dr. Knight, a literary man, whose character was deservedly esteemed, but who, by some speculations in mining, had become embarrassed in circumstances, he is supposed to have afforded aid to the amount of a thousand pounds. We shall not pause to calculate the total amount of his bounties, which have been estimated at so high a sum as two hundred thousand pounds; but it is evident that his generosity knew no other limit than his means.

In 1763, he was elected a fellow of the Royal Society. His reputation soon extended to other countries; he was one of the earliest members of the American Philosophical Society, instituted at Philadelphia. Linnæus distinguished by his name a species of *Polyandria Dygynia*. The Royal Society of Medicine, at Paris, chose him as an associate in 1776; and his letters of admission were the more honourable, because they included a request, that Fothergill would nominate any persons of his acquaintance whom he might deem eligible to become corresponding members of the body. Vicq d'Azyr communicated this mark of confidence in a Latin letter.

When the House of Commons was informed of the fatality of the gaol distemper, which had appeared among the French and Spanish prisoners confined at Winchester, an application was made to him for his opinion, and he recommended Dr. Carmichael Smith as medical superintendent of the prison. A singular success attended the efforts of that accomplished physician, which reflected honour on both parties.

Far from confining his investigations to his own professions, or to natural history, his mind embraced, with great activity, every scheme connected with public improvement. Internal commerce, police, the economy of prisons, all occupied his attention, as the occasions arose for their improvement: he directed his thoughts at one time to the establishment of public baths and of public cemeteries, the latter of which admit, at this moment, of much improvement, and in their present condition, in London, are equally offensive to the eye, as they are probably injurious to the health, of the inhabitants. He was very instrumental in establishing an excellent seminary for the children of Quakers, not in affluent circumstances, at Ackworth. The disputes between the mother country and the American colonies engrossed him very earnestly: he engaged actively in various attempts to promote concord; and appears to have been employed, to a certain extent, in political negociation. Franklin, with whom he treated on this subject, declares, that he doubts whether any man has ever existed more worthy than Fothergill of universal veneration.

Perhaps we might have spoken more largely of his literary essays, which were numerous; in recording the generality of men, these would occupy us more fully, but in the life of Fothergill they form only a secondary consideration. The fame of authorship, and the accomplishments of the man of science, are consumed in the blaze of that exalted virtue which was not contented merely to discharge with indifference the decencies of life, nor even honourably to fulfil its duties, but sought, in every period of its career, to improve the condition of mankind, to befriend the weak, and to feed the hungry, and literally considered the fruits of its own labour as a treasure invested for the benefit of others.

In December 1780, he experienced a second attack of suppression of urine; two years previously it had been relieved, but no art could now remove it. The pain was very acute, his thirst was insatiable, but his mind was as serene as in his best days; he endeavoured even to assume the cheerfulness which was natural to him when in health. He expressed to a friend his hope "that he had not lived in vain, but in degree to answer the end of his creation, by sacrificing interested considerations, and his own ease, to the good of his fellow-creatures." In a fortnight he breathed his last. More than seventy carriages followed his remains to the grave,—not filled with the careless attendants on the great, who mourn in order to be visible, but with individuals whom he had contributed to render happy.

Dr. Hird has drawn, from affectionate intimacy, a personal sketch of this great man, which our readers will view with the gratification which attends a visit to the private apartment of the illustrious. The person of Dr. Fothergill was of a delicate, rather than of an extenuated make; his features were all character; his eye had a peculiar brilliancy of expression, yet it was not easy so to mark the leading trait as to disengage it from the united whole. He was remarkably active and alert, and, with a few exceptions, enjoyed a general good state of health. He had a peculiarity of address and manner, resulting from person, education, and principle; but it was so perfectly accompanied by the most engaging attentions, that he was the genuine polite man, above all forms of breeding. At his meals he was remarkably temperate; in the opinion of some, rather too abstemious, eating sparingly, but with a good relish, and rarely exceeding two glasses of wine at dinner or supper; yet, by this uniform and steady temperance, he preserved his mind vigorous and active, and his constitution equal to all his engagements.

Not satisfied with the acts of generosity which he had multiplied during his lifetime, he directed in his will that the presents which had been made to him should be restored to their donors.—*Family Library.*

---

### BIOGRAPHICAL SKETCH OF THE CELEBRATED GALL.

Jean Joseph Gall was born in 1758, at Tiesenbrunn, in Wurtemburgh, and died at Mont Rouge, near Paris, in the summer of 1829. His father, who was a merchant, sent him, whilst yet very young, to one of his uncles in the duchy of Baden, to commence his education. From this place Gall went to Strasbourgh for the purpose of studying medicine; then to Vienna, where he assumed the garb of the physician, which profession he practised up to 1805, when he left the last-named city to return to his father, who had a desire to see him previous to his death, and also to make a tour in the north of Germany, where he commenced teaching his new doctrine. Finally, he arrived at Paris in 1808, where he continued till his death to devote himself to the practice of medicine, and to promulgate the results of his laborious researches.

From an attentive examination of the head of this celebrated man, who for intellectual capacity may be ranked with the first of the age, the author of the treatise before us drew the following indications, namely: among the organs most developed may be enumerated all those situated on the anterior and superior parts of the forehead, such as the spirit of induction, that of wit, the faculty of abstracting and generalizing, but, above all, benevolence. On the crown and sides of the head, there was also strong developements of firmness or perseverance, caution and cunning, or rather finesse and ingenuity. The accusation of duplicity with which he had been charged, our author regards as unfounded. The sexual appetite was strongly marked upon the occiput, whilst the anterior and inferior parts of the forehead exhibited small indications of the memory of facts and philology. Finally, colour, music, mathematics, mechanics, and especially poetry, were very faint: and this last sense to such a degree that he actually had an antipathy for versification. All the other organs were in a state of ordinary developement. That of locality, which had appeared very prominent, was only a contraction of the skin, produced by the habit of thinking.

To this cranioscopy must be added a strong constitution, a degree of corpulence, and a stature considerably over the mean height. His movements displayed more gravity and energy than lightness and promptitude, his looks much fixedness and penetration. His countenance was sometimes marked with care, and had generally an expression rather of seriousness than gaiety. Calm and circumspect, he was always free from blustering and foolish mirth. A sarcastic smile, mingled with an air of irony, sometimes sprung from his mouth and the alæ of the nose. He had a superb forehead, and a chin slightly prominent, full and firm; a clear skin and fresh complexion, thick lips, and passions more profound than violent. The expression of thought was always clear, precise, often picturesque, but sometimes scornful. His lectures ordinarily consisted of a simple exposition of facts; but, in conversation and discussion, interrogation and irony were most conspicuous. The motions of his extremities and attitudes of his body were very negligent; but the tones of his voice, accent, movements of the head, and physiognomy, were very expressive. In fine, a certain fund of German goodnature redeemed some rather rude expressions of humour, which were neither sufficiently mild nor innocent not to produce a slight irritation.

The cranium, after death, having been carefully separated with a saw from above the eyelids, was found of great thickness (about three lines,) as well as hardness. Between the dura mater and pia mater there were about two ounces of a bloody matter, with some exuberances, one of which was about the size of a pea. The cerebral substance was firm and nearly in a natural condition, although during his illness it had been suspected that the brain was the organ chiefly affected. The skull-cap and brain having been removed, weighed together four pounds, one drachm and a half; the skullcap itself weighed one pound, five ounces, one drachm. Thus the proper weight of the brain alone, when disengaged from its meninges, was two pounds, eleven ounces, and half a drachm; a weight indicating a brain, the dimensions of which are very near the maximum they ever attain.

From all this our author remarks, "It is evident that, in the sense which he attached to the word philosophy, Gall had a head eminently philosophic. He was, in fact, skilful in distinguishing prejudices from eternal truths. He possessed an astonishing perspicacity for penetrating into things, and exhibiting them in a point of view fruitful in useful resources; but, in my opinion, he wanted several faculties to constitute a mind of the order of Descartes, of Newton, of Leibnitz, of Wolf, &c. perhaps even of Bacon. With him, in fact, the faculties of casuality and comparison were well developed, but this was not sufficient to enable him to arrive at a system of philosophy at once severe and positive, which embraced at the same time every thing relative to man, and the chain of admirable phenomena which constituted the moral and physical order of the universe. Many organs, especially those of the mathematics, arts, localities, &c. were too weak in him to admit of his elevation to such a height; but he had the organization which qualified him to lay close hold on human nature, and lay the foundation of the true philosophy of man. Many others, with fewer titles to our gratitude, have covered themselves with immortal glory."—*American Journ. of Med. Sc.* From the "Précis Analytique et Raisonnê du Systeme de Dr. GALL." Paris, 1829.

### METEOROLOGICAL JOURNAL,

By Messrs. Harris and Co. Mathematical Instrument Makers, 50, High Holborn.

| | Moon. | Rain gauge. | Thermom. | | | Barometer. | | DeLuc's Hygrom. | | Winds. | | Atmospheric Variations. | | |
|---|---|---|---|---|---|---|---|---|---|---|---|---|---|---|
| | | | 9 a.m. | max. | min. | 9 a.m. | 10p.m. | 9 a.m. | 10p.m. | 9 a.m. | 10p.m. | 9 a.m. | 2 p.m. | 10p.m. |
| July 20 | | .06 | 65 | 69 | 59 | 29.96 | 29.97 | 57 | 56 | SW | WSW | Cloudy | Sho'ry | Fine |
| 21 | | | 68 | 71 | 62 | 30.04 | 30.06 | 54 | 56 | WSW | WSW | Fine | Fine | Fine |
| 22 | | | 66 | 71 | 63 | .07 | .00 | 57 | 56 | WSW | SW | Cloudy | —— | —— |
| 23 | | | 70 | 72 | 64 | 29.96 | 29.96 | 56 | 54 | WSW | W | Fine | Cloudy | Cloudy |
| 24 | | | 72 | 75 | 67 | .97 | 30.00 | 54 | 50 | W | WSW | Fine | Fine | Fine |
| 25 | | | 71 | 77 | 66 | 30.10 | .10 | 52 | 50 | WSW | WSW | —— | —— | —— |
| 26 | | | 73 | 83 | 68 | .10 | .10 | 51 | 49 | WSW | SW | —— | —— | —— |
| 27 | ☽ | | 75 | 81 | 63 | .16 | .19 | 45 | 48 | SSW | E | —— | —— | —— |
| 28 | | | 70 | 82 | 72 | .19 | .12 | 48 | 50 | E | ENE | —— | —— | —— |
| 29 | | | 76 | 82 | 75 | .01 | 29.88 | 50 | 51 | NE | SE | —— | —— | —— |
| 30 | | | 80 | 81 | 69 | 29.80 | .77 | 51 | 50 | SE | SSW | —— | —— | —— |
| 31 | | | 71 | 79 | 67 | .89 | .89 | 48 | 45 | SSW | WSW | —— | —— | —— |
| Aug. 1 | | | 74 | 77 | 61 | .80 | .72 | 45 | 48 | SSW | SW | —— | —— | —— |
| 2 | | .23 | 65 | 70 | 58 | .67 | .74 | 50 | 51 | WSW | WSW | Cloudy | —— | —— |
| 3 | | | 62 | 67 | 62 | .83 | .91 | 51 | 50 | SW | SW | Fine | —— | Rain |
| 4 | ○ | | 65 | 72 | 65 | .91 | .79 | 50 | 48 | SSW | SSE | Show'y | —— | Cloudy |
| 5 | | | 69 | 74 | 59 | .77 | .85 | 46 | 47 | W | W | Fine | —— | Fine |
| 6 | | | 65 | 67 | 57 | .85 | .86 | 47 | 48 | WNW | W | —— | —— | —— |
| 7 | | | 63 | 68 | 56 | .77 | .65 | 48 | 46 | W | SW | —— | —— | Cloudy |
| 8 | | | 62 | 66 | 59 | .56 | .60 | 49 | 50 | E | E | Rain | —— | Fine |
| 9 | | | 62 | 70 | 56 | .66 | .60 | 50 | 52 | E | NNE | Fine | —— | —— |
| 10 | | .25 | 58 | 70 | 58 | .49 | .48 | 52 | 52 | NE | NE | Cloudy | —— | —— |
| 11 | ☾ | | 62 | 71 | 58 | .54 | .53 | 53 | 52 | NE | S | Foggy | —— | Cloudy |
| 12 | | | 66 | 69 | 58 | .58 | .66 | 52 | 53 | SW | SSW | Cloudy | —— | Cloudy |
| 13 | | | 58 | 62 | 53 | .54 | .64 | 54 | 54 | SSW | WSW | Rain | Rain | Fine |
| 14 | | .38 | 60 | 65 | 52 | .72 | .39 | 53 | 58 | SW | WSW | Fine | Rain | Sho'ry |
| 15 | | .62 | 58 | 66 | 54 | .60 | .72 | 60 | 58 | W | W | Cloudy | Fine | Fine |
| 16 | | | 60 | 60 | 50 | .77 | .81 | 54 | 53 | W | W | Fine | Rain | Fine |
| 17 | | | 58 | 60 | 49 | .81 | .85 | 53 | 53 | W | NW | —— | Fine | Rain |
| 18 | ● | | 54 | 61 | 48 | .96 | 30.00 | 52 | 52 | NW | NW | —— | —— | —— |
| 19 | | | 57 | 60 | 55 | 30.01 | 29.92 | 50 | 53 | NNW | NW | —— | —— | —— |

The quantity of Rain fallen in July was 1 inch and 48·100ths.

